# A pilot, open labelled, randomised controlled trial of hypertonic saline nasal irrigation and gargling for the common cold

**DOI:** 10.1038/s41598-018-37703-3

**Published:** 2019-01-31

**Authors:** Sandeep Ramalingam, Catriona Graham, Jenny Dove, Lynn Morrice, Aziz Sheikh

**Affiliations:** 1 Department of Laboratory Medicine, Royal Infirmary of Edinburgh, 51 Little France Crescent, Edinburgh, EH16 4SA UK; 2 Wellcome Trust Clinical Research Facility, University of Edinburgh, Western General Hospital, Crewe Road South, Edinburgh, EH4 2XU UK; 3 Centre of Medical Informatics, Usher Institute of Population Health Sciences and Informatics, The University of Edinburgh, Medical School Doorway 3, Teviot Place, Edinburgh, EH8 9AG UK

## Abstract

There are no antivirals to treat viral upper respiratory tract infection (URTI). Since numerous viruses cause URTI, antiviral therapy is impractical. As we have evidence of chloride-ion dependent innate antiviral response in epithelial cells, we conducted a pilot, non-blinded, randomised controlled trial of hypertonic saline nasal irrigation and gargling (HSNIG) vs standard care on healthy adults within 48 hours of URTI onset to assess recruitment (primary outcome). Acceptability, symptom duration and viral shedding were secondary outcomes. Participants maintained a symptom diary until well for two days or a maximum of 14 days and collected 5 sequential mid-turbinate swabs to measure viral shedding. The intervention arm prepared hypertonic saline and performed HSNIG. We recruited 68 participants (2.6 participants/week; November 2014-March 2015). A participant declined after randomisation. Another was on antibiotics and hence removed (Intervention:32, Control:34). Follow up data was available from 61 (Intervention:30, Control:31). 87% found HSNIG acceptable, 93% thought HSNIG made a difference to their symptoms. In the intervention arm, duration of illness was lower by 1.9 days (p = 0.01), over-the-counter medications (OTCM) use by 36% (p = 0.004), transmission within household contacts by 35% (p = 0.006) and viral shedding by ≥0.5 log_10_/day (p = 0.04). We hence need a larger trial to confirm our findings.

## Introduction

The common cold is a viral upper respiratory tract infection (URTI). Adults and children get 2-3 and 6-7 attacks respectively of URTI annually^1,2^. In 2016 the UK lost 34.0 million work days (33.1% of total) due to minor illnesses such as URTI^3^. An episode of URTI cost €266.41, €273.36 in Cardiff and Southampton respectively^4^. In the US, 72% of respondents had URTI in the past year costing $40 billion annually^2^. Outbreaks of respiratory tract infections are common in hospitals and care homes with significant morbidity and mortality^5^. URTI can lead to lower respiratory tract infections (LRTI) such as pneumonia, or cause exacerbations in individuals with asthma, chronic obstructive pulmonary disease (COPD), and cystic fibrosis^6^. Since URTI precedes LRTI, early intervention could prevent these complications.

At present, there are no antiviral agents to treat the common cold. Though rhinovirus is called the “common cold virus”, a large number of viruses cause URTI^7^. Hence, specific antiviral treatment is impractical, and we need an intervention effective against multiple viruses. Jalaneti (cleaning the nasal passages with salt water), an ancient practice from India is recommended in Yogic texts for the common cold^8^. A significant reduction in sore throats and colds was reported when Australian wood-workers performed Jalaneti for a year^9^. Three randomised controlled trials (RCT) report the efficacy of salt water for acute URTI. Adam *et al*. reported the lack of improvement in symptoms or duration of illness in adults with a common cold or bacterial rhinosinusitis [hypertonic saline (HS)/normal saline (NS) sprays thrice/day vs. standard care]. However, individuals with a common cold who received HS sprays said they would use it again (p = 0.007)^10^. Sea-water sprays (six-times/day) significantly reduced sore throat, nasal secretions, decongestant/mucolytic use in children with URTI^11^. A Cochrane review concludes that the evidence is very limited^12^. In a recent report, both NS and sea-water drops (thrice/day) reduced the severity of URTI symptoms in young children^13^.

Inhibition of viral replication in the presence of chloride/halide salts was reported in the 1960’s^14^. We have recently reported laboratory evidence that non-myeloid cells (e.g. epithelial, fibroblast and hepatic cells) have an innate immune mechanism, which is augmented in the presence of salt (NaCl)^15^. In cell culture models, DNA, RNA, enveloped and non-enveloped viruses are all inhibited in the presence of NaCl^15^. The antiviral effect is dependent on the availability of chloride ions (and not sodium ions)^15^. In the presence viral infection and the availability of NaCl, cells utilise the chloride ions to produce hypochlorous acid (HOCl)^15^. Since HOCl is the active ingredient in bleach, which is known to have an antiviral effect, the mechanism could be augmented by supplying chloride ions through NaCl to treat infections. Here, we report the results of the Edinburgh and Lothians Viral Intervention Study (ELVIS), a pilot RCT of hypertonic saline nasal irrigation and gargling (HSNIG) versus standard care in adults with URTI to determine if we can recruit and retain participants in Edinburgh and to get initial information on acceptability, duration of symptoms, and viral shedding.

## Results

**Figure 1:**
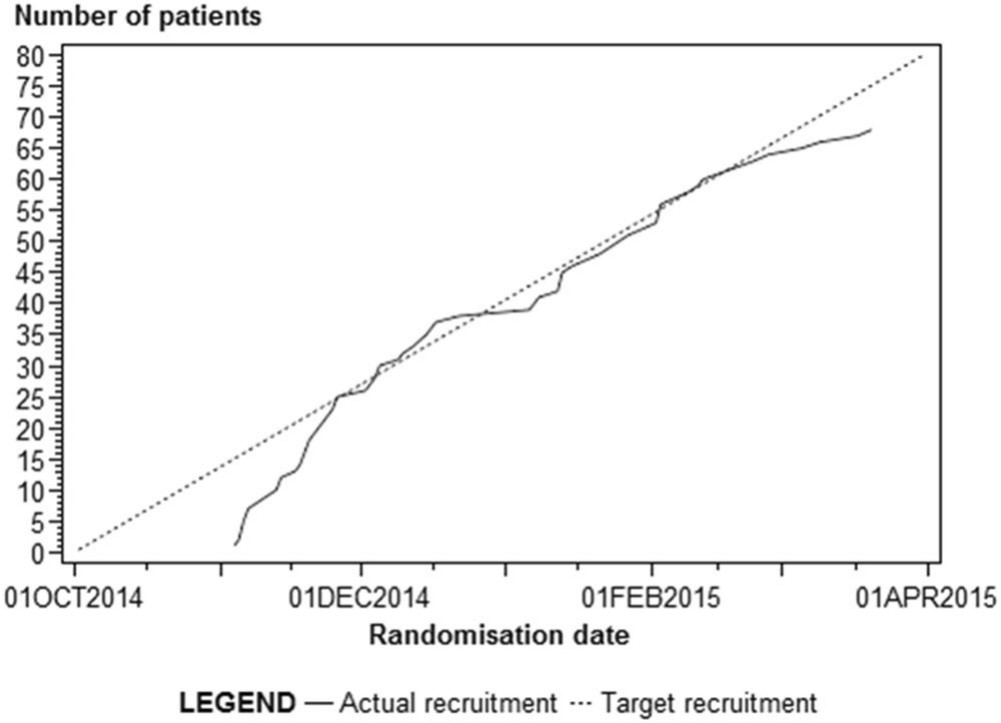
Expected and actual recruitment: Recruitment of participants from the 1st October 2014 till close of recruitment on 31st March 2015. If we take the full 26-week period, this would result in an average of 2.6 participants per week over the study period. However, if we were to take the first and last recruitment dates this give a period of 20 weeks which results in an average of 3.4 participants per week.

**Figure 2:**
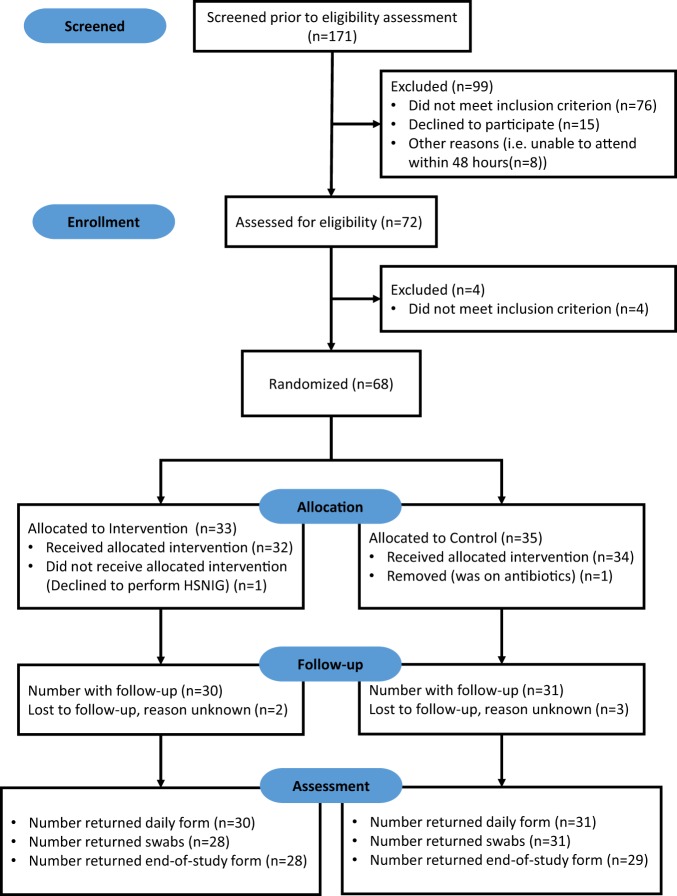
Consort Flow diagram: Based on the “CONSORT extension for Pilot and Feasibility Trials Flow Diagram” (http://www.consort-statement.org/downloads/extensions - Accessed 04/12/2018).

We recruited 68 participants over 26 weeks between October 2014 and March 2015 (2.6 participants/week: Fig. 1). We excluded two (n = 66) [one declined after randomisation, and the other was on antibiotics] and randomised 32 to the intervention arm and 34 to the control arm (Fig. 2). The majority (76%) were women. Most (76%) preferred paper forms over online feedback. Most (Intervention: 94%, Control: 91%) returned the daily forms, end-of-study form (Intervention: 88%, Control: 85%) and swabs (Intervention: 88%, Control: 91%). Of the sixty-six participants, five did not return daily forms. Of the sixty-one, four did not return end-of-study forms. Of the fifty-seven, three did not return samples. All forms and samples were hence available in fifty-four individuals.

80% in the intervention arm used 3% HS, while 9% each used 2.5%, 2% HS. All but one used Cornish sea salt. One participant left without the sea salt and used another brand available at home. An individual in the control arm reported performing nasal irrigation but provided no further information.

The baseline characteristics, symptom severity and degree of interference with daily life were similar in both arms (Table 1). We identified an aetiology in the baseline sample of 73% (48/66) of participants (Table S1). Amongst these, 56% were rhinovirus and 31% were coronaviruses (COV), with the rest due to enterovirus, influenza A virus, parainfluenza virus type 3 (PIV-3), respiratory syncytial virus (RSV) and human metapneumovirus (HMPV). We detected dual infections of rhinovirus with an enterovirus/COV HKU1 in two.

**Table 1:** Baseline characteristics.

	Intervention (n = 32) n (%)	Control (n = 34) n (%)
Age mean(SD)	34.6 (9.3)	39.4 (10.9)
WURSS-21 Score mean (SD)	41.6 (18.2)	43.9 (21.8)
EQ-VAS Score mean(SD)	65.9 (13.6)	63.7 (17.4)
Sex (Female)	24 (75)	25 (74)
Tobacco smoker - current	1 (3)	3 (9)
Tobacco smoker - ex	5 (16)	11 (32)
e-Cigarette smoker - current	0 (1)	1 (3)
e-Cigarette smoker - ex	2 (6)	0 (0)
Adults at home = 1	6 (19)	5 (15)
>1	26 (81)	29 (85)
Children at home = 0	19 (59)	19 (56)
1	7 (22)	5 (15)
>1	6 (19)	10 (29)
No one unwell before them at home	19 (59)	21 (62)
Employment status: Full-time	20 (63)	21 (62)
Part-time	7 (22)	5 (15)
Education: Full time	4 (13)	4 (12)
Other	1 (3)	4 (12)

WURSS: Wisconsin upper respiratory symptom survey; EQ-VAS: EuroQol-Visual Analog Scale.

**Figure 3:**
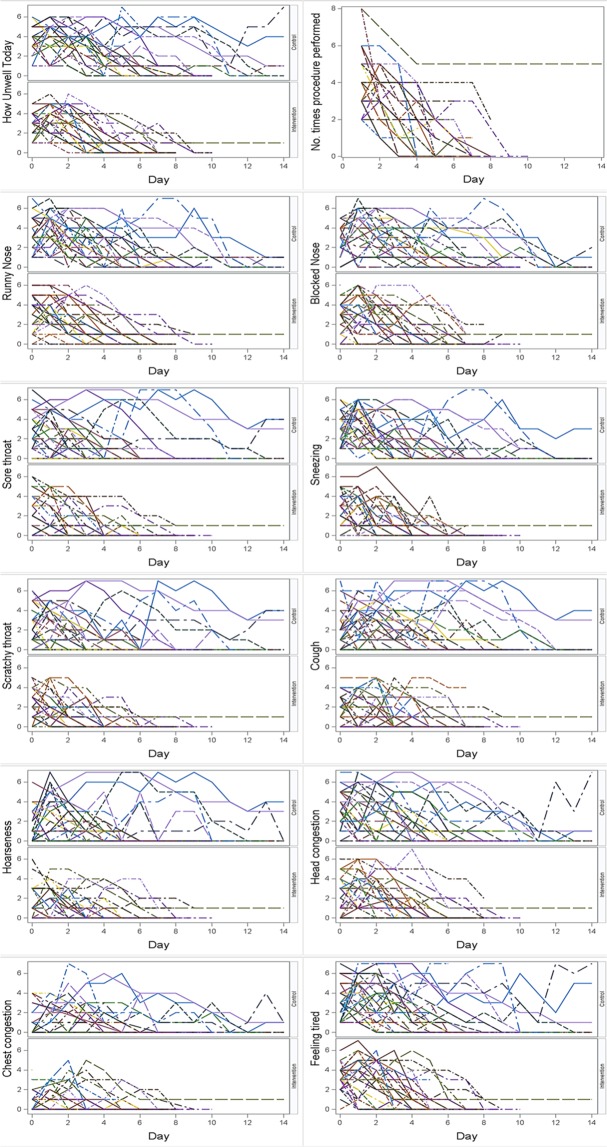
Response from participants over the study period: Each line represents response of a participant over 14 days. Data is shown by treatment group. The global severity question was “How unwell do you feel today”. The responses were graded from 0 (Not unwell), 1 (very mildly), 3 (mildly), 5 (moderately) and 7 (severely unwell). Likewise, each symptom was graded 0 (no symptom) to 7 (severe).

Most participants completed the “WURSS-21-Scot” daily until they were well (i.e. a score of 0) on two days (please see Fig. S1 for daily forms). Intervention and control arms completed the symptom diary for a mean (SD) of 6.8 (2.2) and 8.7 (3.3) days respectively. The intervention arm hence had a reduction in duration of illness by 1.9 days (95% CI = 0.4 to 3.3) (p = 0.01) (Fig. 3). The duration of illness was significantly lower (p = 0.01) even if the first day participants felt well was the end-point [Mean (SD) Intervention: 6.0 (2.4); Control: 8.0 (3.4) days]. Participants performed HSNIG for a median of 5 days (IQR: 3 to 6) and at a median of thrice a day (IQR: 2 to 3) (Fig. 3).

There was a significant reduction in the duration of runny nose (1.8 days, 95% CI:0.4 to 3.2, p = 0.01), blocked nose (2.7 days, 95% CI:1.2 to 4.1, p < 0.001), sneezing (1.5 days, 95% CI:0.3 to 2.9, p = 0.02), cough (2.4 days, 95% CI:0.9 to 4.0, p = 0.003), hoarseness of voice (1.7 days, 95% CI:0.2 to 3.1, p = 0.02) (Fig. 3; Supplementary Table S2).

We could calculate the average WURSS-21 score and EQ-VAS scores from the diaries (please also see supplementary results). The median (IQR) average WURSS 21 score in the intervention group was 13.2 (7.6 to 16.4) [n = 30] and 16.9 (9.9 to 24.7) [n = 31] in the control arm (p = 0.09). The mean (SD) average quality of life measure (EQ-VAS) over the study duration was higher at 74.3 (12.1) [n = 30] for the intervention and 70.8 (15.5) [n = 31] in the control arm. The difference in means of 3.4, 95% CI for difference (−3.7 to 10.6) was not significant (p = 0.338) (Supplementary Table S3).

**Figure 4:**
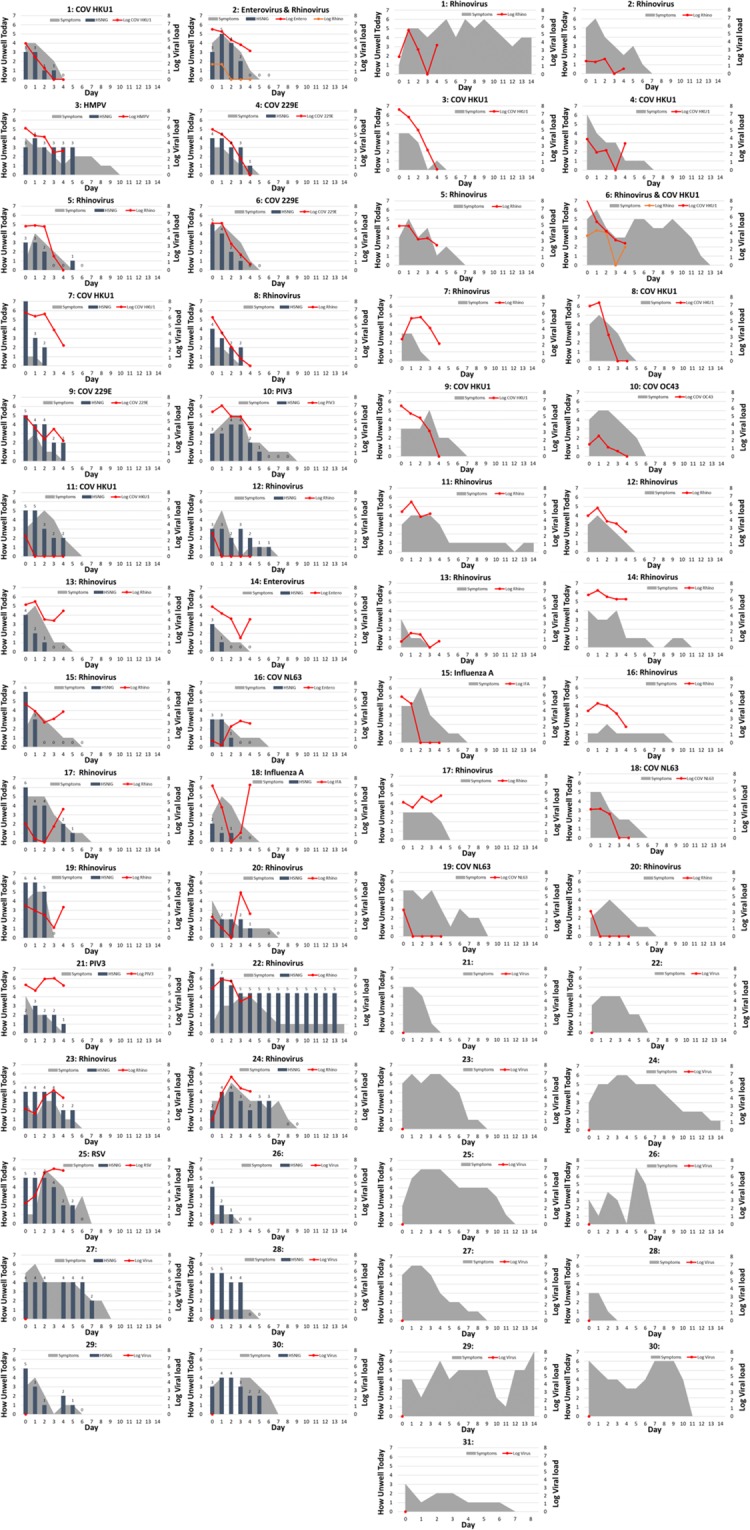
Severity of symptoms, viral shedding and relationship to HSNIG and participant number in each arm: For each participant, the daily scores for the global question ‘how unwell do you feel today’ over 14 days are represented by the grey background. For the intervention arm (shown on the left), the blue columns represent the number of times HSNIG was done that day. For both arms, change in viral shedding (as log values; Red line) is shown for the corresponding days.

Excluding those with no virus detected in the baseline sample [Intervention: 5 (16%); Control: 12 (35%)], samples on days 1–4 to estimate viral shedding was available in 25 and 20 individuals in intervention and control arms respectively (Fig. 4). In four individuals [a COV and a rhinovirus each per arm], no virus was detectable on days 1–4. Details of symptom severity, viral shedding and HSNIG are in Fig. 4.

The median (IQR) reduction in viral shedding between baseline and end-point sample in the intervention arm was −2.23 log_10_ (−3.04 to −0.32) [n = 26] and −1.51 log_10_ (−3.30 to −0.55) [n = 21] in the control arm (p = 0.9). (see supplementary results). We then estimated the proportion of individuals with viral shedding by ≥0.5 log_10_/day between arms (see supplementary results). Four individuals in the intervention arm had paramyxovirus infection but none in the control arm. As paramyxoviruses have a longer life cycle^16,17^, incubation period^18^, duration of viral shedding^19^ and illness^19,20^ compared to rhinovirus^21–24^, the data was analysed after removing these individuals. A higher proportion in the intervention arm had a fall ≥0.5 log_10_/day compared to controls [Intervention 73% [n = 16/22], Control 43% [n = 9/21], difference −30%, 95% CI for difference in proportion (−58 to −2) p = 0.04].

End-of-study forms were available from fifty-seven individuals (Table 2). Over-the-counter medications use (OTCM) use was 36% lower in the intervention arm (95% CI 14 to 59) (p = 0.004). Amongst participants who were not living alone, 35% fewer individuals in the intervention arm (95% CI 10 to 60) had household contacts developing URTI after them (p = 0.006).

**Table 2:** Feedback from participants at the end of study.

	Intervention		Control		p
	n = 28	%	n = 29	%	
Took time off work	3	11	7	24	0.297*
Days off work: 1	2	7	2	7	
2	1	4	1	3	
3	—	0	4	14	
Took over-the-counter medication	14	50	25	86	0.004*
Attended GP	—	0	2**	7	
Attended hospital	1**	4	—	0	
Household contact developed symptoms after participant	8/26	31	19	66	0.006
Performed procedure	28	100	1	3	
Collecting swabs: Easy	19	68	18	62	
Moderate	7	25	3	10	
Difficult	2	7	8	28	
Returning swabs: Easy	26	93	28	97	
Diary completion: Easy	25	89	28	97	
Form completion: Easy	25	89	27	93	
Pre-study information – Useful	27	96	28	97	
Instruction pack – Helpful	28	100	27	93	
HSNIG video: Helpful	27	96	—	—	

*Using fishers exact test due to small counts or expected counts, **Reason for attending GP/hospital was not available, GP: General practitioner, HSNIG – Hypertonic saline nasal irrigation and gargling.

We received feedback on HSNIG from 28/32 participants (Table S4). 93% said that HSNIG made a difference to their symptoms. They found HS easy to prepare (100%). Most preferred to prepare solution in bulk in a flask (86%) and preferred using a small bowl to perform HSNIG (75%). Most considered the procedure either comfortable or moderately comfortable (89%) and the majority (86%) were happy to perform the procedure outside home. The most commonly reported effects were that it helped clear the nose/reduced stuffiness (n = 16), made it easier to breathe (n = 7), speeded up resolution of symptoms (n = 5), reduced the severity of the illness (n = 3) and improved sleep (n = 2). 60% of participants were likely to use HSNIG in the future and 25% of participants were undecided if they would use HSNIG in the future. The figure increased to 86% if the procedure could be made more convenient. Most (71%) however said they were unlikely to use HSNIG as a prophylactic measure.

## Discussion

Our pilot RCT confirms that it’s possible to recruit and retain participants for a full trial of HSNIG with 3% HS. HSNIG reduced the duration of illness (22%), OTCM use (36%) and illness in household members (35%). When individuals infected with similar viruses (rhinovirus, coronavirus, enterovirus and influenza virus) were compared, 30% more individuals had reduction in viral shedding by ≥0.5 log_10_ per day in the intervention arm. This could explain both the reduction in the duration of illness and transmission to household contacts in the intervention arm. However, though the difference between the baseline and end-point samples was larger in the intervention arm than the control arm, the difference was not significant (although this study was not powered to detect differences in these measures). In participants who stopped HSNIG before day four, 54% (7/13) had an increase in viral shedding. There was also an increase/stabilisation of symptoms in 50% (8/16) before symptoms resolved. In fact, four individuals felt the need to restart HSNIG for one or more days (Fig. 4). These finding along with the lower rate of symptomatic household contacts in the intervention arm suggest that HSNIG helps reduce viral replication. Since viruses are shed during breathing and speaking^7^, measure that helps reduce viral shedding would help reduce transmission.

Recruitment was relatively easy though it involved regular email reminders. Advertising through social media could potentially help in future studies. A major concern was whether the population in Edinburgh would be happy to perform HSNIG. Surprisingly, only one individual declined to participate having met the trial nurse. Patient reported compliance with HSNIG was excellent. Participants performed HSNIG more times in the earlier part of the URTI and fewer times as symptoms improved, a trend which was in keeping with the severity of illness (Fig. 3). A surprisingly high proportion (86%) reported performing HSNIG outside their homes. 93% found HSNIG useful and 61% said they would perform HSNIG again if they had a cold, with a higher uptake if the procedure was more convenient. Alternatives such as nasal sprays are options, though they would not have the physical rinsing component of nasal irrigation. Hence a study to compare the two methodologies would be useful.

WURSS 21 score was not significantly different between the two arms, probably a reflection in the sample size. Neither was the EQ VAS score different between arms. EQ VAS score is an indicator of how a person feels on a given day and is not specific to URTI. It is hence probably not suitable for studies on URTI.

Our study has limitations. As a pilot with a primary outcome of establishing if a trial using HSNIG is viable, the study is not powered for efficacy end-points. We hence need a larger trial to confirm our findings. The lack of a placebo group is another limitation. Since our hypothesis was that the chloride ion has an antiviral effect, we were unable to use NS as a control as it could cause a reduction in symptoms. This is supported by results from earlier studies. For e.g. in a cross over trial (10 weeks twice daily nasal spray and 10 weeks without sprays with a two-week washout period), twice a day saline sprays significantly reduced nasal symptoms in military recruits compared to those with no intervention (p = 0.027). The number of episodes of URTI was lower in the period when saline sprays were used compared to the period when sprays were not used. However, the difference was just short of significance (p = 0.05)^25^. Our rationale also seems vindicated by a recent report suggesting that both sea water drops and saline drops were equally effective in treating children <2 years of age with URTI compared to symptomatic controls^13^. Sodium bicarbonate, though commonly used, is uncomfortable in the author’s personal experience. Recent evidence suggests that sodium ion also has an antimicrobial effect^26,27^. Hence, until a safe and comfortable placebo that contain neither chloride, halide or sodium ion is identified, placebo-controlled trials cannot be done.

Another aspect that needs to be considered is the potential benefits of the simple process of flushing in the intervention arm. Even in the presence of a placebo arm, this cannot be answered. Further studies with different methodologies for supplying NaCl (e.g. hypertonic saline sprays, or aerosolised NaCl) may help answer this question.

In the absence of a placebo, we focused on viral shedding as an objective measure of antiviral activity due to HSNIG. There were more individuals without a detectable virus in the baseline sample in the control arm (12/34:35%) compared to the intervention arm (5/32:16%). This difference did not however reach statistical significance (p = 0.059) (Table S1). Though allergic rhinitis (history of allergy with current eye/nose itching or sneezing) was an exclusion criterion, it is possible that some of these individuals could have allergic rhinitis. Or, the infective aetiology might not have been detected in the nose swab. For e.g. sore throat was often recorded by those who did not have a detectable virus. As we collected a nose swab, it is possible that the aetiology could be picked up by including a throat swab along with a nose swab.

Viral shedding is difficult to quantify due to the variability in sampling and as most routine respiratory PCR’s are qualitative assays. We used self-collected mid-turbinate swabs (Copan, Italy) both for participants convenience (no gag reflex) and as the swabs are designed with a stop which increases safety and should help reduce variation in sampling^28^. Since nasal irrigation could physically wash off the virus, we collected swabs first thing in the morning before HSNIG. We used eNAT, a transport medium that inactivates viruses and in which samples are stable at room temperature for at least two weeks. In our hands, samples in eNAT were stable for at least a week at room temperature and could be posted back to the laboratory for testing^29^. Where we identified a virus in the baseline sample, we tested all five samples in the same run to minimise inter-assay variability. To compare viral shedding, we converted CT values to log_10_ values. The baseline samples were hence tested on two occasions. The inter-assay variation between the two results was very low [mean (SD): 0.21 log_10_ (1.17)]. The cut-off of ≥0.5 log_10_ per day used to determine reduction in viral shedding is more than double that of the inter-assay variation seen and hence is unlikely to be an artefact of the testing process. All these measures together have helped produce viral shedding data that could be compared between arms. In four individuals with a positive baseline sample, follow-up samples were negative. In two individuals a sample with a low CT (i.e. high viral copy) were followed by samples with undetectable virus. Since our consent did not include human DNA testing, we could not test for housekeeping genes and cannot be certain if these samples were properly collected. This need to be addressed in future studies.

We detected viruses in 73% of individuals, much higher than 40–55% reported by others^30–32^. This could be due to sampling within 48 hours of onset of illness. Though rhinoviruses and coronaviruses were the commonest, our study confirms that numerous viruses cause URTI. The viral load of the initial sample varied between individuals and sequential sampling is important to detect change in viral shedding. A larger study would help determine the relative efficacy of HSNIG against different viruses.

At baseline, those infected with a virus other than rhinovirus had more individuals with runny nose and blocked nose (data not shown). Sore throat was often recorded in those without a viral aetiology. Hence a baseline throat swab for both bacteria and viruses may help determine the aetiology in these individuals.

The results of ELVIS are significantly different to that from Adam *et al*. which had many methodological issues. They compared 2% HS spray, NS spray (two squirts, thrice a day) and a control group in individuals with the common cold or rhinosinusitis^10^. Though sample size was similar to ELVIS (35–43/arm), individuals were recruited up to 3 weeks after illness onset. Very few had the common cold (12–17/arm), most had bacterial rhinosinusitis and 98% of them were treated with antibiotics. Despite these shortcomings, individuals with the common cold who received HS sprays said they would use it again (p = 0.007). To avoid these shortcomings, we selected only those with a common cold within 48 hours of onset, and who were not on antibiotic therapy.

Strengths of the study are the use of WURSS-21, a validated symptom score diary^33^, for up to two weeks, and sequential sampling over 5 days in otherwise healthy adults. With positive results in a controlled population, we can also look at more challenging population groups in subsequent studies. An alternative strategy would be to use HSNIG as a prophylactic tool. Wood workers who performed nasal irrigation twice a day for a year had fewer episodes of sore throat and colds^9^. Sea water sprays thrice a day, for 12 weeks in children significantly reduced reported illness, school absence and use of medication^11^. Though feedback regarding the use of HSNIG as a prophylactic tool was negative in our population, it may not be reflective of a population at high-risk for complications such as those with asthma/COPD.

Compliance was excellent in our study. We had online videos for preparation of hypertonic saline, performing HSNIG and collection of swabs both for providing instruction to participants and as a handy reminder if needed later on. Participants were all encouraged to prepare the solution and perform HSNIG in the presence of the trial nurse which we believe helped with compliance. Participants were also trained to collect the nose swab by the trial nurse. Hence a pragmatic approach (i.e. patient reported compliance) can be taken to reduce the burden to the participant and the cost of the study. However, in patient groups where compliance might be an issue, compliance could be improved by using video monitoring of the procedures (HSNIG and collecting nasal swabs) via smart phones with support from the clinical team similar to the approach used for tuberculosis treatment^34,35^. The amount of salt used could also be measured at the end of the study. Tests for human DNA could be done to determine if swabs were actually collected before being introduced into the transport medium. Online data entry could be encouraged which would help reduce missing/incorrect data. Reminder messages (by text or email) could be sent to prompt regular data entry and return of samples. However, these decisions would need to be taken considering the population, the burden to the participant and the cost involved.

In this pilot, HSNIG significantly reduced the duration of URTI, OTCM use and illness within the household. A greater fall in viral shedding possibly explains the reduction in duration of symptoms and in symptomatic household contacts. This is in keeping with the lab evidence that cells utilise NaCl to mount an antiviral effect. A larger study powered for clinical and virological end-points is urgently needed to confirm these findings.

## Methods

### Study Setting and Design

We obtained ethical permission from the South-East Scotland Research Ethics Committee (13/SS/0079) and carried out the study in accordance with the Declaration of Helsinki (Clinical trials registration: NCT02438579, May 8, 2015). ELVIS was a pilot unblinded RCT of HSNIG to determine recruitment in Edinburgh as the primary outcome. Acceptability and compliance with HSNIG, quality of life, duration of symptoms and viral shedding were secondary outcomes. With a sample size of 27 per group we would be able to express the proportion of those who return the symptom score diary and samples within that group to within ±19% based on a two-sided 95% confidence interval around an expected proportion of 0.5. With the two groups combined [i.e. a sample size of 54] we would be able to express a proportion to within ±13%. To allow for dropouts we planned to recruit up to 80 participants to have at least 30 participants per arm for analysis. The study was advertised through schools, libraries and general practices in Edinburgh and Midlothian areas, local newspaper coverage, emails within NHS Lothian, online, social media and the study website (www.elvisstudy.com).

#### Identification of individuals with a cold

Individuals with URTI were identified as done by Barrett *et al*.^33,36^ Volunteers had to (1) answer “Yes” to “do you have a cold?” or “do you think you are coming down with a cold?”; (2) have at least one of first four symptoms: nasal discharge, nasal obstruction, sneezing, sore throat, headache, malaise, chilliness and cough and (3) have a Jackson Score of ≥2. Onset of URTI >48 hours, concurrent antibiotic use, pregnancy, known chronic conditions, immunosuppression, allergic rhinitis, inability to perform HSNIG and taking part in another medical trial were exclusion criterion. Volunteers met a trial nurse at a Clinical Research Facility at the Royal Infirmary of Edinburgh (RIE) or the Western General Hospital where informed consent was obtained, and participants were then randomised. Participants were centrally allocated into intervention and control arms using a minimisation algorithm containing sex and smoking status (current/not a current smoker) with a built-in random component to ensure allocation concealment.

#### Feedback

Participants had to maintain a daily form (Fig. S1) until they recorded “not unwell” (i.e. score of 0) on two consecutive days or for a maximum of 14 days or until the individual needed further treatment for URTI and then filled the end-of-study form (Fig. S2). The short form of the Wisconsin Upper Respiratory Symptom Survey (WURSS-21) was used to collect daily symptom data^33^. For use in the Scottish context, and with the authors consent, the words “sick” and “plugged” were replaced with “unwell” and “blocked”, respectively (WURSS-21-Scot) (Fig. S1). Participants were asked to answer the global severity question: “How unwell do you feel today?” which was scored from 0 (not unwell) to 7 (severely unwell). If they scored >0, then symptoms and functional ability were graded 0–7. They then answered the global change question “Compared to yesterday, I feel my cold is” which was scored from 0 (very much better) to 6 (very much worse). Total WURSS-21 score was calculated by adding the scores for all except the first and last question. To calculate the mean WURSS-21 score, the scores for each participant were added and divided by 14. A mean value of EQ-VAS was calculated for each participant over the time questionnaires were returned as it was possible that participants may not score 100 even when a person’s cold symptoms have completely resolved. Both arms documented OTCM use and if they contacted the general practitioner (GP)/nurse for further management of their URTI. Feedback about trial procedures, acceptability, health service use, costs to the patient and suggestions for improving the study and information on symptomatic household contacts were collected at the end of the study. Participants had the option to fill the daily and end-of-study forms online or on paper and return them to the laboratory.

#### Study Procedures

Intervention arm were taught to prepare the hypertonic saline, perform HSNIG and documented the number of times/day and side-effects. They could either prepare 100 ml of hypertonic saline for a single use or prepare in bulk in a clean flask for use during the day. Instructions on how to make the solution and perform HSNIG are in Supplementary Methods and in www.elvisstudy.com. Control arm managed URTI as they normally did. As the hypothesis was chloride ion mediated antiviral effect, normal saline could not be used as a placebo. As sodium bicarbonate was uncomfortable in the authors personal experience, we opted for not including a placebo arm. The rationale for not including a saline placebo arm is elaborated in the discussion. Participants documented OTCM use and were asked to contact to their GP if unwell. The trial nurse helped participants in the intervention arm identify the highest concentration of HS they were comfortable with (from 3%, 2.5%, 2.0% and 1.5%). Intervention arm were taught how to prepare HS and perform HSNIG with videos (www.elvisstudy.com) and given the opportunity to perform HSNIG under supervision. Cornish sea salt, digital-measuring spoon, bowls and flask were provided with instructions to perform HSNIG as many times as required (expected frequency up to 6 times/day for the first two days, reducing in frequency from day 3 as symptoms improved).

### Mid-turbinate swab collection

The trial nurse collected a mid-turbinate swab (day 0) and taught participants on how to collect samples first thing in the morning on days 1–4 (before HSNIG was performed that day in the intervention arm). Flocked mid-turbinate swabs and eNAT transport medium (Copan, Italy) and Royal Mail Safebox were provided with instructions to package and return the samples to the Department of Laboratory Medicine, RIE for testing. Instructions on how to collect and return mid-turbinate swabs are in Supplementary Methods.

#### Virological testing and quantification

Mid-turbinate swabs were tested by an in-house polymerase chain reaction (PCR). The panel included, influenza virus A&B, respiratory syncytial virus (RSV), parainfluenza viruses (PIV) 1–3, human metapneumovirus (HMPV), adenovirus, rhinovirus, enterovirus, parechovirus, bocavirus, coronaviruses (COV) OC43, NL63, 229E, HKU1, and mycoplasma. Day 0 samples were initially tested. Where an agent was identified, all samples (day 0 – day 4) were tested in parallel and the cycle threshold value (CT value) converted to log_10_ to estimate change in viral shedding. CT was capped at 40 as samples with CT values above this level was unlikely to be positive on repeat testing. CT values were converted to log_10_ using this formula (40-CT of sample)/3.3 (a CT of 3.3 represents a log change in viral load by PCR). Since the baseline sample was tested on two separate occasions, reproducibility of testing was assessed by comparing the two results. Inter-assay variation was expected to be <0.5 log_10_. Where consecutive samples had undetectable virus, the first sample that was undetectable was treated as the end-point for viral shedding analysis. For the intervention arm, since NaCl could have an antiviral effect, the day with the lowest shedding when HSNIG was being performed was treated as the end-point. Where HSNIG was stopped earlier than 4 days, since swabs were collected first thing in the morning, the day after HSNIG was stopped was considered the end-point. Hence for the intervention arm, the end-point was day 4 [apart from participant numbers 1, 5, 7, 13, 16, 17, 18 and 19 where it was day 3; 2 (rhinovirus), 14, and 15 where it was day 2; and 11 and 12 where it was day 1]. Two individuals had dual viral infections in their specimen. Both viruses were included for viral shedding analysis.

To determine if there was reduction in viral shedding, the log_10_ value of the day 0 sample was subtracted from the log_10_ value of the end-point. Negative values indicate a reduction in viral shedding and positive values indicate an increase in viral shedding. To determine the reduction in viral shedding per day, these values were divided by the number of days of follow-up. The proportion of individuals with reduction in viral shedding by ≥0.5 log_10_/day was then calculated.

### Statistical analysis

We used SAS v9.4 software for statistical analysis. For categorical data, we present numbers and percentages. For continuous data, we present mean (standard deviation) or median (interquartile range (IQR)) as appropriate. Binomial test for the comparisons of proportions was used to examine differences in proportions and presented along with 95% CI for differences in proportions or chi-square tests depending on the number of groups. To compare differences between treatment arms, two-sample t-tests or Mann-Whitney tests were used, as appropriate.

Participants were considered in the groups to which they were randomised irrespective of treatment received for analysis. Due to the way the data were collected, baseline information was available for all participants. Any subsequent information was the result of participants returning the diary card, potential non-return of diary cards was accounted for by increasing our sample size to allow for drop-outs. In the cases where diary cards were not returned, we do not have any information beyond baseline and therefore we have not used any methods to impute any missing data.

## Supplementary information

Supplementary information
