# Supplementary material for: A pilot, open labelled, randomised controlled trial of hypertonic saline nasal irrigation and gargling for the common cold
Source: Sci Rep. 2019 Jan 31;9:1015. doi: 10.1038/s41598-018-37703-3 (PMC6355924; doi:10.1038/s41598-018-37703-3)
Supplement: Supplementary file 1 — Supplementary information [file 41598_2018_37703_MOESM1_ESM.pdf]

Supplementary material:

Title:

A pilot, open labelled, randomised controlled trial of hypertonic saline nasal irrigation and gargling for the common cold.

Authors:

Sandeep Ramalingam[1*], Catriona Graham[2], Jenny Dove[1], Lynn Morrice[3], Aziz Sheikh[3].

[1] Department of Laboratory Medicine, Royal Infirmary of Edinburgh, 51 Little France Crescent, Edinburgh EH16 4SA.

[2] Wellcome Trust Clinical Research Facility, University of Edinburgh, Western General Hospital, Crewe Road South, Edinburgh, EH4 2XU

[3] Centre of Medical Informatics, Usher Institute of Population Health Sciences and Informatics, The University of Edinburgh, Medical School Doorway 3, Teviot Place, Edinburgh, EH8 9AG

***Corresponding author:**

Dr. Sandeep Ramalingam,

Consultant Virologist, Department of Laboratory Medicine, Royal Infirmary of Edinburgh, 51 Little France Crescent, Edinburgh EH16 4SA.

Email: sandeep.ramalingam@nhslothian.scot.nhs.uk

Supplementary Methods:

INSTRUCTIONS ON THE PREPARATION OF HYPERTONIC SALINE: (FOR VIDEO: WWW.ELVISSTUDY.COM)

Edinburgh & Lothians Viral Intervention Study

Preparation of hypertonic saline:

You are provided two graduated bowls (120ml/250ml) and a flask. You can prepare the solution either in the bowl or in the flask. The instructions are given below.

To make 100ml of 3% solution in the bowl:

- Add 3g of sea salt with digital measuring spoon into a clean bowl.
- Add boiled cooled water to 100 ml mark of bowl (warmth – to suit you). If you add freshly boiled water instead, wait for it to cool before using.
- Mix to ensure salt dissolves.
- Taste the solution. It should taste salty and NOT too salty or sting your mouth
  - If it is too salty or stings your mouth, it will sting your nose.
  - If it is too salty, use the highest concentration you are comfortable with.
  - See table below for lower concentrations.
  - Document which concentration you decide to use.
- The table below gives the amount of salt that needs to be added to make solutions of different strength or volumes.

To make larger volumes of hypertonic solution for use during the day:

- To make a 3% solution, add 15g of sea-salt into 500 ml flask provided.
- Fill the flask to the top with boiling water. Close, shake to mix and then store.
- Prior to performing HSNIG, pour out required quantity into clean bowl.
- Wait for it to cool and perform HSNIG.
- Use a clean flask to prepare solution. Make a fresh batch at least every 24 hours.
- Refer to table below to prepare different concentrations. Please document which concentration you decide to use.

Table:

Amount of Salt in grams to be added to make different volumes of solution:

	Concentration of solution			
Add g of salt to make solution	3.0%	2.5%	2.0%	1.5%
100ml – Bowl	3g	2.5g	2g	1.5g
200ml – Bowl	6g	5g	4g	3g
500ml – Flask	15g	12.5g	10g	7.5g

How to use the digital measuring spoon:

- **To switch on**: Press the **"On/Off/Tare"** button.
- Check if it is measuring in **grams**. (If it says Oz, press **"Mode"** to change to **g**).
- **Tare:** Press the "On/Off/Tare" button to bring reading to 0.0g
- Scoop required amount of salt gently.
- If you need more, scoop required quantity
- If in excess, gently remove excess.
- Measure out salt into bowl / flask as appropriate.
- **To switch off:** Press and hold "On/Off/Tare" button.

Caution:

- Avoid rough handling of the scale. Avoid shaking / dropping scale.
- Do not use the digital measuring spoon to mix the solution.

Edinburgh & Lothians Viral Intervention Study

How to perform hypertonic saline nasal irrigation and gargling (HSNIG):

- Use a **clean** bowl to perform HSNIG.
- Either prepare the solution in a clean bowl or transfer from flask into a clean bowl.
- Wait for solution to cool.
- Bend over the sink, close right nostril.
- Bring bowl to the left nostril such that the solution is in contact with the left nostril.
- Inhale **slowly and steadily** through the nose. The solution will enter the nose, and go to the back of the throat. Do not swallow the solution.
- Some solution will come out via the nose and some via the mouth (keep mouth open).
- Repeat this procedure three times for each nostril.
- If the solution that drains is not clear, repeat the procedure for each nostril until it is clear.
- Then gargle three times with the remainder of the solution.
- Clean the bowl with soap and water and store.
- Blow each nostril gently into a tissue (First with head held straight and next bent over sink with chin close to chest to make excess fluid come out. Keeping your chin close to chest will assist any liquid which may have entered the sinuses drain).
- For the next 10-15 minutes, you may feel the need to blow your nose a few times.
- After the procedure, your symptoms should decrease. However, symptoms may re-appear (within 1-2 hours in the first few days and within 4-5 hours after a few days of irrigation). Irrigate as soon as you feel symptoms returning.
- Hence for the first two – three days, you may have to do the procedure for around 6 times a day. This should decrease in subsequent days.
- If you wake up in the middle of the night with symptoms, try to perform the procedure as it may help you sleep better.

COLLECTING NASAL SWABS AND RETURN OF SPECIMEN:

Edinburgh & Lothians Viral Intervention Study

Collecting nasal swabs and return of specimen:

On day 0: In the presence of the trial nurse.

- Remove the swab from the packaging
- Insert the swab into a nostril (left or right) up to the swab's collar.
- Rotate three times.
- Insert the swab into the transport medium **marked day 0**
- Snap the top part of the swab (it will break in the middle where you can see a line).
- Replace the cap and close the transport medium tight.
- Hand over the swab to the trial nurse.

On days 1-4: At home.

- The nasal swab has to be collected first thing in the morning.
- Please collect the swab before performing nasal irrigation + gargling if you are in the intervention arm
- Follow instructions to collect swab as given above
- On day 1, insert the swab into the transport medium marked day 1
    - On day 2, insert the swab into the transport medium marked day 2
    - On day 3, insert the swab into the transport medium marked day 3
    - On day 4, insert the swab into the transport medium marked day 4
- Snap the top part of the swab (it will break in the middle where you can see a line).
- Replace the cap and close the transport medium tight.
- Place the sample within a pocket in the absorbent material. Place it in the plastic self-seal bag.
- Store the sample in a safe place at room temperature.
- On day 4, place all samples in the self-seal plastic bag along with the absorbent material and seal the bag.
- Place the self-seal bag in clear plastic container in the Royal Mail Safebox™(Step 3– Instructions on using the Royal Mail Safebox)
- Remove the card board separator from the lid and dispose of it (Step 5– Instructions on using the Royal Mail Safebox)
- Press the lid over the top of the container and firmly press shut (Step 6– Instructions on using the Royal Mail Safebox)
- Peel the outer backing from the self-adhesive label. Then wrap around the Royal Mail Safebox™ (Step 7/8- Instructions on using the Royal Mail Safebox)
- Drop of the Royal Mail Safebox™ in any post-box (Step 9– Instructions on using the Royal Mail Safebox).

Supplementary Results:

Pattern of completion of daily forms: Most participants completed the "WURSS-21-Scot" daily until they were well (i.e. a score of 0) on two days. Five individuals completed the daily forms for fourteen days but did not score 0 on two consecutive days. Eleven participants stopped before scoring 0 on 2 consecutive days. Of these, eight scored 0 on the last day of completion, two scored 1 on two consecutive days and the last person stopped on day 6 with a score of 5.

WURSS-21 and EQ-VAS Scores: From the "WURSS-21-Scot" diaries, the average WURSS-21 score could be calculated for each participant over 14 days. Of the 549 "WURSS-21-Scot" diaries, a daily score was not available for 34 records [7=no information, 9 = 0 recorded for 'how unwell today' and no further questions answered, 18 where questions in the WURSS-21-Scot were missed]. The median (IQR) average WURSS 21 score in the intervention arm was 13.2 (7.6, 16.4) [n=30] and 16.9 (9.9, 24.7) [n=31] in the control arm (p=0.09). The median average scores and ranges for individual symptoms over 14 days are given in Table S3. Head congestion (p=0.04) and sneezing (p=0.03) were significantly lower in the intervention arm. The mean (SD) average quality of life measure (EQ-VAS) over the study duration was higher at 74.3 (12.1) [n=30] for the intervention arm and 70.8 (15.5) [n=31] in the control arm. The difference in means of 3.4, 95% CI for difference (-3.7, 10.6) was not significant (p=0.338). As EQ-VAS, is an indicator of how a person feels on a given day, is not specific to URTI, it is probably not suitable for studies on URTI.

Change in viral shedding a symptom severity on days after HSNIG was stopped (Intervention arm): Thirteen individuals stopped nasal irrigation before day four followed by an increase in viral shedding is seen in seven (participants 13-19), and stabilisation of symptoms in eight

(participant numbers 13-18, 27, 29). Four individuals restarted HSNIG for one or more days (participant numbers 5, 17, 27, 29) while in four, symptoms stabilised for 2-3 days before becoming asymptomatic (participant numbers 13-16).

Difference in viral shedding between arms: To determine if there was a difference in viral shedding between arms, we deducted the baseline value from the end-point sample. A negative value means a reduction in viral shedding and a positive value means an increase in viral shedding. The median (IQR) reduction in viral shedding in the intervention arm was -2.23 $\log_{10}$ (-3.04, -0.32) [n=26] and -1.51 $\log_{10}$ (-3.30, -0.55) [n=21] in the control arm. The reduction in viral shedding between arms was however not statistically significant (p=0.9).

Difference in reduction in viral shedding/day between arms: Next the proportion of individual with reduction in viral shedding by ≥0.5 $\log_{10}$/day was calculated. When all samples were included for this calculation, there was no difference between the two arms in the proportion of individuals who had a fall ≥0.5 $\log_{10}$/day [Intervention arm 65% [n=17/26], Control arm 43% [n=9/21], difference -23%, 95% CI for difference in proportion (-50, -5) p=0.114]. However, four individuals in the intervention arm had paramyxovirus infection (Figure 4; 3=HMPV; 10&21=PIV-3; 25=RSV), but there were no paramyxovirus infections in the control arm. Since this could potentially affect the results (as paramyxoviruses tend to have a longer life cycle [17,18], incubation period [19], duration of viral shedding [20] and illness [20,21] compared to rhinovirus [22-25]), we reanalysed the data having removed individuals infected with paramyxoviruses. Now, a higher proportion of the intervention arm had a fall ≥0.5 $\log_{10}$/day compared to controls [intervention arm 73% [n=16/22], control arm 43% [n=9/21], difference -30%, 95% CI for difference in proportion (-58, -2) p=0.04].

Supplementary tables and figures:

Table S1: Viruses identified in the day 0 samples.

	Intervention (n=32)		Control (n=34)	
	n	%	n	%
Rhinovirus	14	44	13	38
All Coronaviruses (COV)	7	22	8	24
COV-229E	3	9	0	0
COV-OC43	0	0	1	3
COV-HKU1	3	9	5*	15
COV-NL63	1	3	2	6
Enterovirus	2*	6	1**	3
Influenza A virus	1	3	1	3
Respiratory syncytial virus	1	3	1**	3
Parainfluenza virus type 3	2	6	0	0
Human metapneumovirus	1	3	0	0
Negative	5	16	12	35

The panel included influenza A, B, respiratory syncytial virus, parainfluenza viruses 1-3, rhinovirus, enterovirus, parechovirus, COV 229E, COV OC43, COV NL64 and COV HKU1, human metapneumovirus, adenovirus, bocavirus and mycoplasma

*- 1 dual infection with rhinovirus.

** - No daily forms or follow up samples received.

Table S2: Days for individual symptoms to get better and functional questions to normalise:

Well / Symptoms	Mean days		Mean Difference	95% CI Mean		P value
	Intervention (n=30)	Control (n=31)				
Days to feeling well	6.8	8.7	1.9	0.4	3.3	**0.01**
Runny nose	6.7	8.5	1.8	0.4	3.2	**0.01**
Blocked nose	6.0	8.7	2.7	1.2	4.1	**<0.001**
Sneezing	5.7	7.3	1.5	0.3	2.9	**0.02**
Sore throat	5.1	6.4	1.3	-0.2	2.8	0.09
Scratchy throat	5.1	6.3	1.2	-0.2	2.5	0.09
Cough	4.9	7.4	2.4	0.9	4.0	**0.003**
Hoarseness	4.8	6.5	1.7	0.2	3.1	**0.02**
Head congestion	6.4	7.3	0.9	-0.6	2.4	0.26
Chest congestion	4.4	5.2	0.8	-0.6	2.3	0.25
Feeling tired	6.5	7.9	1.4	0.0	2.8	0.06

Table S3: Difference in severity of symptoms between arms:

	Intervention (n=30)		Control (n=31)		p value
	Median	IQR	Median	IQR	
Total WURSS-21 score	13.2	7.6, 16.4	16.9	9.9, 24.7	0.09
Runny nose	1.0	0.6, 1.5	1.4	0.8, 2.1	0.21
Blocked nose	0.9	0.4, 1.4	1.4	0.7, 2.2	0.09
Sneezing	0.6	0.4, 0.9	0.9	0.6, 1.5	**0.03**
Sore throat	0.5	0.1, 0.9	0.6	0.3, 1.4	0.34
Scratchy throat	0.5	0.3, 0.6	0.5	0.1, 1.4	0.94
Cough	0.5	0.1, 1.1	0.9	0.2, 1.9	0.10
Hoarseness	0.3	0.1, 0.8	0.7	0.3, 1.1	0.25
Head congestion	0.7	0.4, 1.1	1.2	0.7, 1.8	**0.04**
Chest congestion	0.2	0.0, 0.4	0.3	0.0, 1.1	0.33
Feeling Tired	1.0	0.4, 1.4	1.3	0.5, 2.1	0.14
	**Mean**	**SD**	**Mean**	**SD**	**p value**
EQ-VAS	74.3	12.1	70.8	15.5	0.338

Table S4: Feedback about HSNIG – Intervention arm:

	n=28	%
Ease of preparing solution: Easy	28	100
Made solution in bulk (Flask)	24	86
Preferred small bowl for HSNIG	21	75
HSNIG was: Comfortable	11	39
Moderately comfortable	14	50
Uncomfortable	3	11
Performed HSNIG outside home	24	86
If so was it easy to do? Easy	8	29
Moderate	11	39
Difficult	5	18
Equipment cleaning: Easy	27	96
HSNIG on the whole: Convenient	11	39
Moderate	14	50
Inconvenient	3	11
Do you feel HSNIG made a difference? Yes	26	93
Would you use HSNIG in the future: Likely	17	61
Undecided	7	25
Unlikely	4	14
If HSNIG was more convenient: Likely	24	86
Undecided	2	7
Unlikely	2	7
Would you use HSNIG for prophylaxis: Likely	6	21
Undecided	2	7
Unlikely	20	71

Supplementary figures:

Figure S1: Daily Form “WURSS-21-Scot”

Edinburgh and Lothians Viral Intervention Study

Subject Number: ____________________ Date: Time:

Daily Form – Day 1

	Not unwell 0	Very Mildly 1	2	Mildly 3	4	Moderately 5	6	Severely 7
1 How unwell do you feel today?	O	O	O	O	O	O	O	O

Once you have answered ‘not unwell’ for 2 consecutive days or for a maximum of 14 days you do not need to complete any further information on the daily form. Please go directly to the ‘End of study form’.

2. Please rate the average severity of your cold symptoms over the last 24 hours for each symptom

	Do not have this 0	Very Mildly 1	2	Mildly 3	4	Moderately 5	6	Severely 7
Runny nose	O	O	O	O	O	O	O	O
Blocked (plugged) nose	O	O	O	O	O	O	O	O
Sneezing	O	O	O	O	O	O	O	O
Sore throat	O	O	O	O	O	O	O	O
Scratchy throat	O	O	O	O	O	O	O	O
Cough	O	O	O	O	O	O	O	O
Hoarseness	O	O	O	O	O	O	O	O
Head congestion	O	O	O	O	O	O	O	O
Chest congestion	O	O	O	O	O	O	O	O
Feeling tired	O	O	O	O	O	O	O	O

3. Over the last 24 hours, how much has your cold interfered with your ability to:

	Not at all 0	Very Mildly 1	2	Mildly 3	4	Moderately 5	6	Severely 7
Think clearly	O	O	O	O	O	O	O	O
Sleep well	O	O	O	O	O	O	O	O
Breathe easily	O	O	O	O	O	O	O	O
Walk, climb stairs, exercise	O	O	O	O	O	O	O	O
Accomplish daily activities	O	O	O	O	O	O	O	O
Work outside the home	O	O	O	O	O	O	O	O
Work inside the home	O	O	O	O	O	O	O	O
Interact with others	O	O	O	O	O	O	O	O
Live your personal life	O	O	O	O	O	O	O	O

4. Compared to yesterday, I feel that my cold is:

Very much better	Somewhat better	A little better	The same	A little worse	Somewhat worse	Very much worse
O	O	O	O	O	O	O

ELVIS Daily Form V1.0 28102014 Page 1 of 42

Edinburgh and Lothians Viral Intervention Study

Subject Number: ____________________ Date: Time:

Daily Form – Day 1

<u>Procedure – **All:**</u>

5.a. Please note you are allowed to either blow or not blow your nose before nasal swab is collected. We just need to know the procedure you followed.

Did you collect nasal swab? Yes O No O

If Yes: Did you blow your nose prior to collecting nasal swab? Yes O No O

<u>Procedure – **Intervention arm only**, controls please go to Q9.</u>

5.b. If you performed the nasal irrigation procedure yesterday did you use the same concentration of solution? Yes O No O Did not perform yesterday O

If yes please go to Q6, if not:

How much salt have you used? ______.____ grams

How much water have you used? ______.____ ml

6. How many times have you performed the procedure in the last 24 hours?

0 O 1 O 2 O 3 O 4 O 5 O 6 O 7 O 8 O 9 O 10 O 11 O 12 O

7. What techniques did you use?

Irrigation + gargle O

Irrigation only O

Gargle only O

None O

If none please indicate reason: ____________________________

8. Did you notice any side effects? Yes O No O

If yes please indicate how severe you felt these:

	None 0	1	2	3	4	Severe 5
Irritation	O	O	O	O	O	O
Burning	O	O	O	O	O	O
Pain	O	O	O	O	O	O
Dryness	O	O	O	O	O	O
Runny nose	O	O	O	O	O	O
Other	O	O	O	O	O	O
If other please describe:						

9. Since you last completed these questions have you taken any cold / flu related medications? Yes O No O

If yes, what did you take? ____________________________

ELVIS Daily Form V1.0 28102014 Page 2 of 42

Edinburgh and Lothians Viral Intervention Study

Subject Number: ____________________ Date: Time:

Daily Form – Day 1

10.

To help people say how good or bad a health state is, we have drawn a scale (rather like a thermometer) on which the best state you can imagine is marked 100 and the worst state you can imagine is marked 0.

We would like you to indicate on this scale how good or bad your own health is today, in your opinion. Please do this by drawing a line from the box below to whichever point on the scale indicates how good or bad your health state is today.

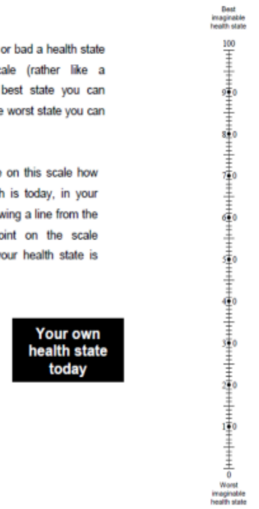

11. Since you last completed these questions have you sought further medical attention for your cold?

Yes O No O

If yes where did you seek help?

GP O

Hospital O

Other O please specify:____________________________

____

____

(If you have answered Yes to this question, please go to the “End of study form”)

ELVIS Daily Form V1.0 28102014 Page 3 of 42

Figure S2: End-of-Study Form

Edinburgh and Lothians Viral Intervention Study

End of Study Form

Subject Number: ____________________ Date: Time:

1. As a result of your symptoms did you:

Take any time off work/education?: Yes ☐ No ☐
If yes please indicate number of days: ___________days

Take any medications? Yes ☐ No ☐
If yes please provide details: ____
____
____

Visit your GP Yes ☐ No ☐
Attend hospital Yes ☐ No ☐

2. Did anyone else in your house experience similar symptoms after you?
Yes ☐ No ☐

If yes, how many adults experienced symptoms _______
How many children experienced symptoms _______

3. Did you perform gargling/nasal irrigation: Yes ☐ No ☐
If no please go to question 6, if yes please complete the following:

If you were allocated to the ***control arm***, i.e. not allocated to perform nasal irrigation / gargling, what method did you use and how did you find out about this method?
____

If you were allocated to the ***intervention arm***,
Which container did you usually make up the solution in?
Small bowl ☐ Large bowl ☐ Flask ☐

Which bowl did you usually use to perform gargling/irrigation?
Small bowl ☐ Large bowl ☐

Please select the box that best describes the following statements:

	Did not perform	Very easy	Easy	Moderate	Difficult	Very difficult
How comfortable was the procedure?		☐	☐	☐	☐	☐
How easy was it to make the solution?		☐	☐	☐	☐	☐
How easy was it to perform outside your home?	☐	☐	☐	☐	☐	☐
How easy was it to clean everything required for the procedure?	☐	☐	☐	☐	☐	☐
How easy was it to carry with you everything required for the procedure?	☐	☐	☐	☐	☐	☐
How convenient did you find the procedure as a whole?	☐	☐	☐	☐	☐	☐

ELVIS End of Study form V1.0, 26/10/2014 1/2

Edinburgh and Lothians Viral Intervention Study

4. Do you feel the procedure has made a difference to your symptoms?

Yes ☐ No ☐

If yes, what difference do you feel it has made: ______________________________
____
____
____

5. Future use of gargling/nasal irrigation:

Please select the box that best describes the following statements:

	Very likely	Likely	Undecided	Unlikely	Very unlikely
If you have the symptoms of the cold in the future, how likely would you be to use this procedure?	☐	☐	☐	☐	☐
If the procedure was more convenient to perform, how likely would you be to use this procedure?	☐	☐	☐	☐	☐
Would you consider performing the gargling/irrigation procedure once or twice a day as a preventative measure?	☐	☐	☐	☐	☐

6. Study procedures and documentation:
Please select the box that best describes the following statements:

	Did not perform	Very easy	Easy	Moderate	Difficult	Very difficult
How easy was it for you to collect the swabs?	☐	☐	☐	☐	☐	☐
How easy was it to return the swabs?	☐	☐	☐	☐	☐	☐
How easy was it to complete the daily diary information?	☐	☐	☐	☐	☐	☐
How easy was it to complete the other forms?	☐	☐	☐	☐	☐	☐

	Not viewed	Very helpful	Helpful	Undecided	Unhelpful	Very unhelpful
How helpful did you find the information provided before joining the study?		☐	☐	☐	☐	☐
How helpful did you find the instructions and information provided in your study pack?		☐	☐	☐	☐	☐
**Intervention group only:** How helpful did you find the instructional video for the irrigation methods?	☐	☐	☐	☐	☐	☐

ELVIS End of Study form V1.0, 26/10/2014 2/2
